# Supplementary material for: Survival disparities and competing mortality risks in offspring of consanguineous marriages in Yemen: A 26-year retrospective cohort analysis
Source: PLoS One. 2026 May 29;21(5):e0349764. doi: 10.1371/journal.pone.0349764 (PMC13221058; doi:10.1371/journal.pone.0349764)
Supplement: S1 File — STROBE and RECORD checklists for observational studies. (DOCX) [file pone.0349764.s001.docx]

File S1: COMPLETE STUDY CHECKLISTS

# 1. STROBE-RECORD CHECKLIST

| **Section** | **Item** | **STROBE Item** | **RECORD Extension** | **Reported** |
| --- | --- | --- | --- | --- |
| TITLE & ABSTRACT | 1 | Design and summary | - | ✓ Title, Abstract |
|  | RECORD 1.1 | - | Data type specification | ✓ Methods: Data Sources |
|  | RECORD 1.2 | - | Codes/algorithms used | ✓ File S2, Tables S1-S3 |
| INTRODUCTION | 2 | Background/rationale | - | ✓ Introduction |
|  | 3 | Objectives | - | ✓ Introduction |
| METHODS | 4 | Design | - | ✓ Methods: Study Design |
|  | 5 | Setting | - | ✓ Methods: Setting |
|  | 6 | Participants | - | ✓ Methods: Cohort Assembly |
|  | RECORD 1.3 | - | Data cleaning methods | ✓ Methods: Quality Assurance |
|  | 7 | Variables | - | ✓ Methods: Diagnostic Criteria |
|  | 8 | Data sources | - | ✓ Methods: Data Sources |
|  | 9 | Bias | - | ✓ Methods: Statistical Analysis |
|  | 10 | Study size | - | ✓ Methods: Sample Size |
|  | 11 | Quantitative variables | - | ✓ Methods: Statistical Analysis |
|  | 12 | Statistical methods | - | ✓ Methods, File S2 |
|  | RECORD 7.1 | - | Missing data handling | ✓ Methods: Missing Data |
|  | RECORD 7.2 | - | Cohort linkage | ✓ Methods: Cohort Assembly |
|  | RECORD 12.1 | - | Sensitivity analyses | ✓ Methods: Sensitivity Analysis |
| RESULTS | 13 | Participants | - | ✓ Table 1 |
|  | 14 | Descriptive data | - | ✓ Tables 1-2, S1-S3 |
|  | 15 | Outcome data | - | ✓ Tables 2-5 |
|  | 16 | Main results | - | ✓ Tables 2-4, Figures 1-3 |
|  | 17 | Other analyses | - | ✓ Supplementary Analyses |
| DISCUSSION | 18 | Key results | - | ✓ Discussion |
|  | 19 | Limitations | - | ✓ Discussion: Limitations |
|  | 20 | Interpretation | - | ✓ Discussion |
|  | 21 | Generalizability | - | ✓ Discussion: Limitations |
| OTHER INFO | 22 | Funding | - | ✓ Funding Section |
|  | RECORD 13.1 | - | Database access | ✓ Data Availability |
|  | RECORD 13.2 | - | Data validation | ✓ Methods: Quality Assurance |
|  | RECORD 13.3 | - | Funding role | ✓ Funding Section |
|  | RECORD 16.1 | - | Data sharing | ✓ Data Availability |
|  | RECORD 16.2 | - | Linkage methods | ✓ Methods: Follow-up |
| ETHICS | RECORD 19.1 | - | IRB approval | ✓ Ethical Approval |
|  | RECORD 19.2 | - | Consent/waiver | ✓ Ethical Considerations |
|  | RECORD 19.3 | - | Confidentiality | ✓ Ethical Considerations |

# 2. DATA QUALITY & VALIDATION CHECKLIST

| **Quality Domain** | **Specific Check** | **Metric/Result** | **Acceptance Threshold** | **Status** |
| --- | --- | --- | --- | --- |
| Data Collection | Interviewer training completion | 100% | 100% | ✓ PASS |
|  | Inter-rater reliability (κ) | 0.84 | 0.80 | ✓ PASS |
|  | Pilot study completion | 50 households | ≥30 households | ✓ PASS |
| Data Entry | Double data entry rate | 20% | ≥10% | ✓ PASS |
|  | Data entry error rate | 0.8% | <2% | ✓ PASS |
|  | Missing data rate (overall) | 2.3% | <5% | ✓ PASS |
| Validation | Medical record concordance | 84.2% | 80% | ✓ PASS |
|  | Temporal consistency checks | 96.7% | 95% | ✓ PASS |
|  | Cross-source validation | 92.4% | 90% | ✓ PASS |
| Biological Plausibility | Age at death distribution | Consistent | No outliers | ✓ PASS |
|  | Birth year range | 1998-2024 | Consistent | ✓ PASS |
|  | Sex ratio | 1.05 (M:F) | 0.9-1.1 | ✓ PASS |
| Logical Consistency | Age death ≤ age last followup | 100% | 100% | ✓ PASS |
|  | Followup date > birth date | 100% | 100% | ✓ PASS |
|  | Consanguinity degree hierarchy | 100% | 100% | ✓ PASS |

# 3. STATISTICAL ANALYSIS VALIDATION CHECKLIST

| **Statistical Assumption** | **Test/Method** | **Result** | **Interpretation** | **Status** |
| --- | --- | --- | --- | --- |
| Proportional Hazards | Schoenfeld residuals (global) | χ²=8.34, p=0.134 | No significant violation | ✓ VALID |
|  | Covariate-specific tests | All p>0.05 | Assumptions met | ✓ VALID |
| Linearity | Martingale residuals | p=0.234 | Linear form adequate | ✓ VALID |
|  | Restricted cubic splines | DF=3, p=0.421 | No improvement | ✓ VALID |
| Influential Observations | Maximum DFBETA | 0.12 | <0.2 threshold | ✓ VALID |
|  | Observations with DFBETA>0.2 | 0 | 0% of sample | ✓ VALID |
|  | Cook's distance | 0.08 | <1.0 threshold | ✓ VALID |
| Multicollinearity | Maximum VIF | 2.34 | <5.0 threshold | ✓ VALID |
|  | Condition number | 18.7 | <30 threshold | ✓ VALID |
| Model Fit | Cox-Snell residuals | R²=0.342 | Adequate fit | ✓ VALID |
|  | Harrell's C-statistic | 0.79 (0.76-0.82) | Good discrimination | ✓ VALID |
|  | Calibration slope | 1.02 (0.95-1.09) | Well calibrated | ✓ VALID |
| Competing Risks | Fine-Gray assumptions | Satisfied | Proportional subhazards | ✓ VALID |
|  | Cumulative incidence | Monotonic | Valid estimates | ✓ VALID |

# 4. ETHICAL COMPLIANCE CHECKLIST

| **Ethical Requirement** | **Documentation** | **Verification** | **Status** |
| --- | --- | --- | --- |
| Institutional Approval | Radfan College University IRB (RUC-IRB-2023-045) | Certificate dated 12 April 2023 | ✓ APPROVED |
|  | Yemeni Ministry of Health (AMREC 2024-011) | Approval letter | ✓ APPROVED |
| Informed Consent | Written consent forms (File S6) | 100% adult participants | ✓ OBTAINED |
|  | Witnessed thumbprint consent | For illiterate participants | ✓ OBTAINED |
|  | Parental permission + child assent | For minors | ✓ OBTAINED |
| Participant Protection | Confidentiality protocol | Anonymization procedures | ✓ IMPLEMENTED |
|  | Risk minimization plan | Bereavement support referrals | ✓ IMPLEMENTED |
|  | Cultural adaptations | Community advisory board | ✓ IMPLEMENTED |
| Data Security | Encryption standards | AES-256 encryption | ✓ COMPLIANT |
|  | Access controls | Two-factor authentication | ✓ COMPLIANT |
|  | Data retention policy | 10 years secure storage | ✓ COMPLIANT |
| Benefit-Risk Balance | Direct benefits | Genetic counseling provided | ✓ PROVIDED |
|  | Indirect benefits | Community health education | ✓ PROVIDED |
|  | Risk assessment | Minimal psychosocial risk | ✓ ACCEPTABLE |

# 5. REPRODUCIBILITY CHECKLIST

| **Reproducibility Element** | **Standard** | **Our Implementation** | **Status** |
| --- | --- | --- | --- |
| Code Availability | Complete analysis code | File S11 (Complete R code) | ✓ FULL |
| Data Availability | Minimal dataset | File S12 (12 representative cases) | ✓ PROVIDED |
|  | Data dictionary | File S13 (Complete specifications) | ✓ PROVIDED |
| Software Environment | Version specification | R 4.2.1, packages listed | ✓ SPECIFIED |
|  | Package versions | survival_3.5-7, cmprsk_2.2-11 | ✓ DOCUMENTED |
| Randomization | Seed setting | set.seed(20241215) | ✓ SET |
| Computational Steps | Raw to results pipeline | Complete script with comments | ✓ DOCUMENTED |
| Decision Documentation | Analytical choices | Decision log in File S2 | ✓ RECORDED |
| Version Control | Code management | Git repository (OSF) | ✓ MAINTAINED |
| Output Verification | Result consistency | Cross-checked outputs | ✓ VERIFIED |

# 6. SENSITIVITY ANALYSIS COMPLETENESS CHECKLIST

| **Sensitivity Scenario** | **Method** | **Primary Result** | **Sensitivity Result** | **Conclusion** |
| --- | --- | --- | --- | --- |
| Missing Data | Multiple imputation (20 datasets) | HR=2.84 | HR=2.79 (2.28-3.41) | ✓ ROBUST |
|  | Pattern mixture models | HR=2.84 | HR=2.81 (2.30-3.43) | ✓ ROBUST |
|  | Complete case analysis | HR=2.84 | HR=2.81 (2.29-3.45) | ✓ ROBUST |
| Time Scales | Age-based (primary) | HR=2.84 | Reference | - |
|  | Calendar time | HR=2.84 | HR=2.86 (2.34-3.50) | ✓ ROBUST |
|  | Time since diagnosis | HR=2.84 | HR=2.89 (2.36-3.54) | ✓ ROBUST |
| Competing Risks Methods | Fine-Gray (primary) | HR=2.84 | Reference | - |
|  | Cause-specific hazards | HR=2.84 | HR=2.88 (2.36-3.52) | ✓ ROBUST |
|  | Cumulative incidence | HR=2.84 | Consistent patterns | ✓ ROBUST |
| Model Specification | Full model (primary) | HR=2.84 | Reference | - |
|  | Reduced model (AIC) | HR=2.84 | HR=2.82 (2.31-3.45) | ✓ ROBUST |
|  | Lasso-penalized | HR=2.84 | HR=2.80 (2.29-3.42) | ✓ ROBUST |
| Confounding Control | Base adjustment | HR=2.84 | Reference | - |
|  | E-value assessment | HR=2.84 | E-value=3.8 | ✓ ROBUST |
|  | Propensity score | HR=2.84 | HR=2.79 (2.28-3.41) | ✓ ROBUST |
| Diagnostic Accuracy | Base classification | HR=2.84 | Reference | - |
|  | 5% misclassification | HR=2.84 | HR=2.79 (2.28-3.41) | ✓ ROBUST |
|  | 15% misclassification | HR=2.84 | HR=2.70 (2.21-3.30) | ✓ ROBUST |

# 7. TRANSPARENCY AND OPENNESS CHECKLIST

| **TOP Guideline** | **Requirement** | **Our Compliance** | **Status** |
| --- | --- | --- | --- |
| Citation Standards | Data citation | OSF DOI: 10.17605/OSF.IO/TKP5X | ✓ COMPLIANT |
|  | Code citation | File S11 with version control | ✓ COMPLIANT |
| Data Transparency | Data availability | Minimal dataset + full code | ✓ COMPLIANT |
|  | Access restrictions | Ethical restrictions documented | ✓ COMPLIANT |
| Analytic Methods | Code availability | Complete R code provided | ✓ COMPLIANT |
|  | Computational details | Software versions specified | ✓ COMPLIANT |
| Research Materials | Materials sharing | All instruments provided | ✓ COMPLIANT |
|  | Protocol availability | Full protocol in supplements | ✓ COMPLIANT |
| Design & Analysis | Preregistration | Not applicable (retrospective) | 🔄 NA |
|  | Analysis plan | File S5 (Statistical plan) | ✓ COMPLIANT |
| Replication | Reproducibility | All elements provided | ✓ COMPLIANT |
|  | Replication materials | Complete package available | ✓ COMPLIANT |

# 8. CONFLICT-AFFECTED RESEARCH ETHICS CHECKLIST

| **Conflict-Specific Consideration** | **Our Approach** | **Documentation** | **Status** |
| --- | --- | --- | --- |
| Safety of Participants | Security assessments, safe locations | Safety protocol in File S2 | ✓ ADDRESSED |
| Safety of Researchers | Team training, communication plans | Field manual in File S4 | ✓ ADDRESSED |
| Informed Consent Quality | Enhanced explanations, no coercion | Consent forms (File S6) | ✓ ADDRESSED |
| Confidentiality in Insecurity | Immediate anonymization, no identifiers | Privacy protocol in File S2 | ✓ ADDRESSED |
| Community Engagement | Advisory board, leader consultations | Meeting minutes in File S14 | ✓ ADDRESSED |
| Adaptive Methodology | Flexible data collection, remote options | Protocol adaptations in File S4 | ✓ ADDRESSED |
| Benefit Maximization | Immediate referrals, community feedback | Benefit documentation in File S6 | ✓ ADDRESSED |
| Ongoing Ethics Review | Continued monitoring, pause protocols | Monitoring reports in File S14 | ✓ ADDRESSED |
| Data Integrity | Multiple verification, conflict-period checks | Quality reports in File S4 | ✓ ADDRESSED |
