# Supplementary material for: Survival disparities and competing mortality risks in offspring of consanguineous marriages in Yemen: A 26-year retrospective cohort analysis
Source: PLoS One. 2026 May 29;21(5):e0349764. doi: 10.1371/journal.pone.0349764 (PMC13221058; doi:10.1371/journal.pone.0349764)
Supplement: S2 File — Detailed methodological protocols including missing data procedures and sensitivity analysis framework. (DOCX) [file pone.0349764.s002.docx]

**File S2: SUPPLEMENTARY METHODS**

**S1. Detailed Statistical Appendix**

**Competing Risks Methodology:**

The Fine-Gray subdistribution hazards model was employed to account for competing mortality risks.The subdistribution hazard function for cause j is defined as:

```

λ_j(t) = lim_{Δt→0} Pr(t ≤ T < t + Δt, ε = j | T ≥ t ∪ (T ≤ t ∩ ε ≠ j)) / Δt

```

where T is the time to event and ε indicates the event type. The risk set includes individuals who haven't experienced any event and those who experienced competing events.

**Model Specification:**

The full Cox proportional hazards model with time-dependent covariates:

h(t|X,Z(t)) = h₀(t) × exp(β₁X₁ + β₂X₂ + ... + βₖXₖ + γ₁Z₁(t) + γ₂Z₂(t))

where X represents Time-independent covariates and Z(t) represents time-dependent covariates.

**Time Scale Specification:**

· Primary time scale: Time since birth (age in years)

· Secondary time scales: Calendar time, time since diagnosis

· Left truncation accounted for late entry into observation

· Right-censoring at study end (December 31, 2024)

**Model Diagnostics:**

· Proportional hazards: Schoenfeld residuals (Global test: χ²=8.34, p=0.134)

· Influential observations: Maximum DFBETA=0.12 (<0.2 threshold)

· Functional form: Martingale residuals assessment

· Multicollinearity: All VIF < 2.5

**S2. Data Collection and Ascertainment Protocols**

**Vital Status Determination:**

1. Household Interviews: Structured interviews with primary caregivers, multiple informant verification (minimum 2 sources), historical event anchoring

2. Medical Record Review: Hospital/clinic records (42% coverage), primary healthcare registries, specialist documentation

3. Community Sources: Mosque records, school registries, community leader documentation

4. Verbal Autopsy: WHO 2016 standards, physician-coded with standardized algorithms, consensus panel for uncertain cases (15%)

**Genetic Disorder Ascertainment:**

· Standardized diagnostic criteria per disorder category

· Age at diagnosis determination through multiple sources

· Severity classification (mild/moderate/severe) based on functional impact

· Comorbidity assessment and documentation

**Quality Assurance:**

· Inter-rater reliability: κ = 0.84 for cause of death coding

· Temporal consistency checks across different time periods

· Cross-source validation between medical records and interview data

**S3. Follow-up Procedures and Quality Assurance**

**Entry and Exit Definitions:**

· Entry time: Date of birth

· Exit time: Date of death, loss to follow-up, or study end

· Right-censoring for survivors at last contact date

**Loss to Follow-up Handling:**

· Active tracking: Multiple contact attempts, alternate informant identification

· Passive surveillance: Community reporting systems, healthcare facility linkage

· Sensitivity analysis: Multiple imputation for missing survival times

**Data Validation:**

· Range checks for biological plausibility

· Logical consistency verification

· Cross-source validation (medical records vs. interview data)

· Expert review of complex cases

**S4. Statistical Analysis Methodology**

**Survival Analysis Framework:**

· Kaplan-Meier: Product-limit method with Greenwood confidence intervals

· Cox models: Multivariable regression with time-dependent covariates

· Stratification by disorder category, consanguinity degree, birth cohort

· Log-rank tests with Sidak adjustment for multiple comparisons

**Competing Risks Analysis:**

· Fine-Gray subdistribution hazards model

· Cause-specific hazards models

· Cumulative incidence function estimation

· Gray's test for comparing cumulative incidence functions

**Covariate Specification:**

Time-independent:Sex, birth order, consanguinity degree, parental education, residence

Time-dependent:Disorder severity progression, healthcare access changes, socioeconomic transitions

Contextual:Birth cohort, geographic region, healthcare infrastructure

Healthcare Access (Time-dependent covariate): Healthcare access was categorized as Low, Medium, or High based on a composite score. This score incorporated: (1) travel time to the nearest primary healthcare center (<30 min = 2 points, 30-60 min = 1 point, >60 min = 0 points); (2) availability of a consistent healthcare provider for the child (Yes=1 point, No=0 points); and (3) reported financial barriers to accessing needed medical care in the past year (No barriers=1 point, Any barriers=0 points). Scores of 0-1 were classified as 'Low', 2 as 'Medium', and 3-4 as 'High' access.

**S5. Sensitivity Analyses Framework**

**Comprehensive Sensitivity Testing:**

1. Different Time Scales: Age-based, calendar time, time since diagnosis

2. Missing Data Handling: Multiple imputation (20 datasets), pattern mixture models

3. Competing Risks Approaches: Cause-specific vs. subdistribution hazards

4. Censoring Assumptions: Administrative vs. informative censoring

5. Cause of Death Misclassification: Simulation-based correction

**Model Specification Sensitivity:**

· Covariate selection: Forward/backward selection, clinical knowledge-based

· Functional form: Linear vs. non-linear terms, interaction inclusion

· Alternative distributional assumptions

**S6. Power and Precision Considerations**

**Retrospective Power Analysis:**

· 90% power to detect hazard ratios ≥1.8 (α=0.05, two-sided)

· Event rate: 18.6% overall mortality

· Adequate power for main cause-specific analyses

· Sufficient power for major subgroups

**Precision Assessment:**

· Confidence interval width for key parameters

· Bootstrap estimation for complex parameters

· Small sample bias correction for rare outcomes

· Empirical Bayes shrinkage when appropriate

**S7. Software Implementation**

**Statistical Environment:**

· R version 4.2.1 with RStudio IDE 2022.07.2

· Core packages: survival, cmprsk, timereg, mice, ggplot2, survminer

**Computational Considerations:**

· Efficient data management with data.table

· Memory optimization for time-dependent data

· Parallel processing for bootstrap and imputation

· Version control (Git) for reproducibility

**Model Convergence:**

· Convergence monitoring for complex models

· Alternative optimization algorithms

· Starting value sensitivity assessment

**S8. Quality Control Measures**

**Pre-Collection Quality Control:**

· 2-week intensive training with certification

· 50-household pilot study

· Inter-rater reliability assessment (κ > 0.80 required)

· Instrument refinement based on pilot results

**During Collection Monitoring:**

· Daily field supervision with random spot-checking

· 10% random back-checking by supervisors

· Real-time data quality monitoring

· Weekly data quality review meetings

**Analytical Quality Assurance:**

· Pre-specified analysis plan

· Blinded analysis when feasible

· Independent statistical code review

· STROBE and RECORD guidelines adherence

**S9. Ethical Considerations and Data Privacy**

**Ethical Approvals:**

· Radfan University College IRB (REF: RUC-IRB-2023-045)

· Yemeni Ministry of Health Research Ethics Committee

· Data Monitoring Committee for longitudinal aspects

**Participant Protection:**

· Written informed consent from all adult participants

· Witnessed verbal consent with thumbprint for illiterate participants

· Parental permission with child assent for minors

· Bereavement support referral pathways

**Data Security:**

· Complete anonymization of personal identifiers

· Encrypted databases with access logging

· Two-factor authentication for data access

· Secure transmission protocols

**S10. Missing Data Handling Protocol**

**Missing Data Patterns:**

· Survival times: 2.3% missing

· Cause of death: 4.7% uncertain

· Covariates: 3.1% incomplete

· Follow-up status: 1.8% unknown

**Multiple Imputation Implementation:**

· 20 imputed datasets using chained equations

· Full conditional specification imputation

· Predictive mean matching for continuous variables

· Rubin's rules for combining estimates

**Sensitivity Analysis:**

· Pattern mixture models for different missing data mechanisms

· Selection models for informative missingness

· Worst-case scenario analysis

· Range of plausible values
