# Supplementary material for: Survival disparities and competing mortality risks in offspring of consanguineous marriages in Yemen: A 26-year retrospective cohort analysis
Source: PLoS One. 2026 May 29;21(5):e0349764. doi: 10.1371/journal.pone.0349764 (PMC13221058; doi:10.1371/journal.pone.0349764)
Supplement: S3 File — Structured interview forms and data abstraction tools. (DOCX) [file pone.0349764.s003.docx]

**File S3: Data_Collection_Instruments**

**DATA COLLECTION INSTRUMENTS**

**A. HOUSEHOLD INTERVIEW SCHEDULE**

**Section 1: Demographic Information**1.1 Household identification: ______
1.2 Interview date:***/***/_____
1.3 Interviewer: ______
1.4 Region: Urban/Rural

**Section 2: Parental Information**2.1 Age of mother: ____
2.2 Age of father: ____
2.3 Consanguinity relationship:
 □ First cousins
 □ Second cousins
□ Other relative (specify: ______)
 □ Non-consanguineous

**Section 3: Reproductive History**3.1 Total pregnancies: ____
3.2 Live births: ____
3.3 Stillbirths: ____
3.4 Children who died: ____

**Section 4: Child-Specific Information**
(Repeat for each child)

4.1 Child ID: ______
4.2 Birth date:***/***/_____
4.3 Sex: Male/Female
4.4 Vital status: Alive/Deceased
4.5 If deceased:

Date of death: ***/***/_____

Age at death: ____ years ____ months

Primary cause of death: ______

Place of death: Home/Hospital/Other
4.6 Genetic disorders diagnosed:
 □ Hematological disorder
 □ Congenital anomaly
□ Neurodevelopmental disorder
 □ Sensory impairment
 □ Other: ______
4.7 Healthcare access:

Distance to nearest clinic: ____ km

Regular healthcare provider: Yes/No

Financial barriers: Yes/No

**Section 5: Socioeconomic Status**5.1 Parental education: Illiterate/Primary/Secondary/University
5.2 Household income category: Low/Medium/High
5.3 Health insurance: Yes/No

**B. MEDICAL RECORD ABSTRACTION FORM**

Patient ID: ______
Facility: ______
Record dates: ______ to ______

**Diagnoses:**

Primary diagnosis: ______

Secondary diagnoses: ______

Age at diagnosis: ______

Diagnostic methods: Clinical/Lab/Genetic/Imaging

**Treatments:**

Medications: ______

Surgeries: ______

Hospitalizations: ______

**C. VERBAL AUTOPSY QUESTIONNAIRE (WHO 2016 STANDARD)**

**Part I: Identification**Deceased ID: ______
Interview date: ______
Respondent relationship: ______

**Part II: Background**

Education: ______

Occupation: ______

**Part III: Signs and Symptoms**

Open-ended narrative: ______

Structured symptom checklist (42 items)

Duration of illness: ______

**Part IV: Physician Coding**

Immediate cause: ______

Underlying cause: ______

Coder confidence: High/Medium/Low

**FULL FORMS AVAILABLE IN SEPARATE ATTACHMENTS**
