# Supplementary material for: Survival disparities and competing mortality risks in offspring of consanguineous marriages in Yemen: A 26-year retrospective cohort analysis
Source: PLoS One. 2026 May 29;21(5):e0349764. doi: 10.1371/journal.pone.0349764 (PMC13221058; doi:10.1371/journal.pone.0349764)
Supplement: S4 File — Complete quality assurance and verification procedures. (DOCX) [file pone.0349764.s004.docx]

**File S4: Quality_Assurance_Protocol**

**QUALITY ASSURANCE PROTOCOL**

**1. PRE-DATA COLLECTION QUALITY CONTROL**

**1.1 Staff Training:**

2-week intensive training program

Certification required for all interviewers

Inter-rater reliability testing (κ > 0.80 required)

Practical field exercises (10 supervised interviews)

**1.2 Instrument Testing:**

50-household pilot study

Cognitive testing of questions

Translation-back translation for Arabic instruments

Cultural adaptation review by local experts

**2. DURING DATA COLLECTION MONITORING**

**2.1 Field Supervision:**

Daily supervisor spot-checking (10% of interviews)

Random back-checking within 48 hours

GPS verification of interview locations

Audio recording review (10% random selection)

**2.2 Data Quality Monitoring:**

Real-time electronic data validation

Range checks for biological plausibility

Logical consistency checks

Missing data tracking and resolution

**3. POST-DATA COLLECTION QUALITY ASSESSMENT**

**3.1 Data Validation:**

Double data entry for 20% of forms

Cross-source validation (medical records vs interviews)

Temporal consistency analysis

Expert review of complex cases

**3.2 Quality Metrics:**

Interview completion rate: 98.2%

Item missing rate: <2%

Inter-rater reliability: κ = 0.84

Test-retest reliability: ICC = 0.91

**4. ANALYTICAL QUALITY CONTROL**

**4.1 Statistical Quality:**

Pre-specified analysis plan

Independent code review

Convergence monitoring for iterative models

Sensitivity analysis framework

**4.2 Documentation:**

Complete audit trail of data transformations

Version control for analytical code

Decision log for methodological choices

**QUALITY ASSURANCE REPORTS GENERATED MONTHLY**
