# Supplementary material for: Survival disparities and competing mortality risks in offspring of consanguineous marriages in Yemen: A 26-year retrospective cohort analysis
Source: PLoS One. 2026 May 29;21(5):e0349764. doi: 10.1371/journal.pone.0349764 (PMC13221058; doi:10.1371/journal.pone.0349764)
Supplement: S5 File — Pre-specified statistical analysis plan. (DOCX) [file pone.0349764.s005.docx]

**File S5: Statistical_Analysis_Plan**

**STATISTICAL ANALYSIS PLAN**

**1. PRIMARY OBJECTIVES**

1.1 Estimate disorder-specific survival functions
1.2 Quantify cause-specific mortality using competing risks
1.3 Evaluate consanguinity degree as independent mortality predictor

**2. ANALYSIS PLAN**

**2.1 Descriptive Analysis**

Frequencies and percentages for categorical variables

Means and standard deviations for continuous variables

Medians and interquartile ranges for skewed variables

**2.1.1 Data Structure Verification:**

- Summary statistics for full cohort (n=3,427)

- Verification of minimal dataset representativeness

- Comparison of key characteristics between minimal and full datasets

- Documentation of any differences in variable distributions

**2.2 Survival Analysis**

Kaplan-Meier estimates with Greenwood confidence intervals

Log-rank tests with Sidak adjustment for multiple comparisons

Cox proportional hazards models with time-dependent covariates

**2.3 Competing Risks Analysis**

Fine-Gray subdistribution hazards models

Cumulative incidence function estimation

Gray's test for comparing cumulative incidence functions

**3. MODEL SPECIFICATION**

**3.1 Primary Cox Model:**
h(t|X) = h₀(t) exp(β₁consanguinity + β₂disorder_type + β₃sex + β₄residence + β₅birth_cohort + γZ(t))

**3.2 Competing Risks Model:**
λ_j(t) = λ_j₀(t) exp(β_j₁consanguinity + ... + γ_jZ(t))

**4. SENSITIVITY ANALYSES**

**4.1 Multiple Time Scales:**

Age-based time scale (primary)

Calendar time scale

Time since diagnosis scale

**4.2 Missing Data Handling:**

Complete case analysis

Multiple imputation (20 datasets)

Pattern mixture models

**4.3 Model Assumptions:**

Proportional hazards assessment (Schoenfeld residuals)

Influential observations (DFBETA statistics)

Functional form (Martingale residuals)

**5. SAMPLE SIZE JUSTIFICATION**

5.**1 Precision and Sample Size Justification**

- Total sample: 3,427 offspring

- 638 events (deaths)

- Precision: 95% CI width for primary HR = 1.12 (HR: 2.32-3.44)

- Event-per-variable ratio: 79.8 events per predictor (exceeds 10-20 threshold)

- Confidence intervals for key parameters exclude clinically unimportant effects

**6. SOFTWARE AND IMPLEMENTATION**

**6.1 Primary Software:**

R version 4.2.1

Key packages: survival, cmprsk, timereg, mice

**6.2 Code Management:**

Git version control

Modular code organization

Comprehensive commenting
