# Supplementary material for: Survival disparities and competing mortality risks in offspring of consanguineous marriages in Yemen: A 26-year retrospective cohort analysis
Source: PLoS One. 2026 May 29;21(5):e0349764. doi: 10.1371/journal.pone.0349764 (PMC13221058; doi:10.1371/journal.pone.0349764)
Supplement: S6 File — WHO 2016 verbal autopsy standard instrument and physician coding algorithms. (DOCX) [file pone.0349764.s006.docx]

**File S6: Verbal_Autopsy_Protocol**

**VERBAL AUTOPSY PROTOCOL - WHO 2016 STANDARD**

**1. INTRODUCTION**

This protocol follows WHO 2016 verbal autopsy standards for cause of death determination in settings without complete medical certification.

**2. TRAINING AND CERTIFICATION**

**2.1 Interviewer Training:**

3-day intensive training on VA instrument

Certification through standardized testing

Minimum κ = 0.75 for cause assignment

**2.2 Physician Coder Training:**

Medical doctors with ≥5 years experience

Training on ICD-10 coding

Certification through test cases

**3. DATA COLLECTION PROCEDURE**

**3.1 Respondent Selection:**

Primary caregiver preferred

Multiple informants encouraged

Minimum mourning period: 2 months

**3.2 Interview Setting:**

Private, comfortable location

45-60 minutes duration

Audio recording with permission

**4. CAUSE OF DEATH DETERMINATION**

**4.1 Physician Coding:**

Two independent physicians

Third arbitrator for discrepancies

ICD-10 codes with confidence levels

**4.2 Automated Methods:**

InterVA-5 algorithm

Tariff 2.0 method

Consensus with physician coding

**5. QUALITY CONTROL**

**5.1 Validation:**

15% random validation with medical records

Inter-rater reliability assessment

Temporal consistency checks

**5.2 Performance Metrics:**

Concordance with medical records: 84.2%

Cohen's κ: 0.79 (95% CI: 0.71-0.87)

Physician agreement: 91.3%

**6. ETHICAL CONSIDERATIONS**

Sensitivity to bereavement

Psychological support referrals

Cultural appropriateness maintained

**APPENDICES:**

Full WHO 2016 VA instrument

Physician coding guidelines

Validation study results
