# Supplementary material for: Survival disparities and competing mortality risks in offspring of consanguineous marriages in Yemen: A 26-year retrospective cohort analysis
Source: PLoS One. 2026 May 29;21(5):e0349764. doi: 10.1371/journal.pone.0349764 (PMC13221058; doi:10.1371/journal.pone.0349764)
Supplement: S7 File — Detailed results for: S1 (Multiple Imputation), S2 (Landmark Analyses), S3 (Cause-Specific vs. Subdistribution Hazards), S4 (Probabilistic Bias Analysis), S5 (Interaction Effects), S6 (Geographic Subgroups), S7 (Economic Burden), S8 (Temporal Trend Validation), S9 (Competing Risks by Birth Cohort), S10 (Model Performance). (DOCX) [file pone.0349764.s007.docx]

**File S7: Supplementary_Analyses_S1-S10**

**SUPPLEMENTARY ANALYSES S1-S10**

**Analysis S1: Multiple Imputation Sensitivity Analysis**

20 imputed datasets using chained equations

Results consistent with complete case analysis

Consanguinity HR: 2.79 (2.28-3.41) vs 2.84 in primary analysis

**Analysis S2: Landmark Analyses**

Landmarks at 1, 5, and 10 years

Consistent consanguinity effects across time points

HR at 1-year landmark: 2.91 (2.35-3.60)

**Analysis S3: Cause-Specific Hazards vs Subdistribution Hazards**

Cause-specific models show similar patterns

Hematological disorders: CSH HR=8.48 vs SDH HR=8.42

**Analysis S4: Probabilistic Bias Analysis for Cause of Death Misclassification**

Multiple scenarios (5%, 10%, 15% misclassification)

Results robust to plausible misclassification levels

Corrected consanguinity HR range: 2.70-2.81

**Analysis S5: Interaction Effects Exploration**

Significant consanguinity × healthcare access interaction (p<0.001)

Modest disorder type × birth cohort interaction (p=0.045)

**Analysis S6: Subgroup Analyses by Geographic Region**

Strongest consanguinity effects in rural remote areas

HR gradient: Urban Central 2.45 → Rural Remote 3.12

**Analysis S7: Economic Burden Detailed Calculation**

Direct medical costs: $12.4 million

Indirect costs: $49.85 million

Total economic burden: $62.25 million

**Analysis S8: Temporal Trend Validation**

Joinpoint regression confirms significant improvements

Annual percent change: 3.2% (95% CI: 2.1-4.3%)

**Analysis S9: Competing Risks by Birth Cohort**

Earlier cohorts: higher competing risks from infections

Recent cohorts: shift to disorder-specific mortality

**Analysis S10: Model Performance Assessment**

C-statistic: 0.79 (0.76-0.82)

Calibration slope: 1.02 (0.95-1.09)

Brier score: 0.16
