# Supplementary material for: Survival disparities and competing mortality risks in offspring of consanguineous marriages in Yemen: A 26-year retrospective cohort analysis
Source: PLoS One. 2026 May 29;21(5):e0349764. doi: 10.1371/journal.pone.0349764 (PMC13221058; doi:10.1371/journal.pone.0349764)
Supplement: S10 File — Comprehensive variable definitions, coding schemes, and measurement units. (DOC) [file pone.0349764.s010.doc]

============================================================

MINIMAL DATASET - DATA DICTIONARY

============================================================

Study: Survival Disparities in Consanguineous Offspring

Full cohort: 3,427 offspring (1998-2024)

Minimal dataset: 12 representative cases

============================================================

VARIABLE DEFINITIONS:

1. id: Unique identifier (1-12 for minimal dataset)

2. sex: Biological sex (Male/Female)

3. birth_year: Year of birth (1999-2018)

4. consanguinity_degree:

- Non-consanguineous

- Beyond second cousins

- Second cousins

- First cousins

5. disorder_type: Major disorder category

- Hematological (blood disorders)

- Congenital anomaly (structural defects)

- Neurodevelopmental (brain development disorders)

- Sensory impairment (hearing/vision)

- No disorder (healthy controls)

6. disorder_subtype: Specific diagnosis

7. vital_status: Alive/Deceased at last follow-up

8. age_death: Age at death in years (blank if alive)

9. cause_death: Primary cause of death (ICD-10 based)

10. residence: Urban/Rural

11. parent_education: Highest parental education level

- Illiterate

- Primary

- Secondary

- University

12. birth_cohort: 5-year birth cohort groups

13. healthcare_access: Composite score (Low/Medium/High)

14. age_last_followup: Age at last contact in years

15. followup_status: Final status (Alive/Deceased)

NOTES:

- Minimal dataset includes representative cases from:

* All birth cohorts (1998-2024)

* All consanguinity degrees

* All disorder categories

* Both vital status outcomes

* Both residence types

* All education levels

- For deceased cases: age_death = age_last_followup

- For alive cases: age_last_followup = age at study end (Dec 2024)

- Full analyses conducted on complete cohort (n=3,427)

============================================================
