# Supplementary material for: Survival disparities and competing mortality risks in offspring of consanguineous marriages in Yemen: A 26-year retrospective cohort analysis
Source: PLoS One. 2026 May 29;21(5):e0349764. doi: 10.1371/journal.pone.0349764 (PMC13221058; doi:10.1371/journal.pone.0349764)
Supplement: S11 File — Navigation guide for all supplementary files and reproducibility instructions. (DOC) [file pone.0349764.s011.doc]

============================================================================

COMPLETE ANALYSIS CODE - USER GUIDE

============================================================================

FILE: File S5_Complete_Analysis_Code.R

PURPOSE: Reproducible analysis for "Survival Disparities in Consanguineous Offspring"

AUTHOR: Research Team

============================================================================

PREREQUISITES:

1. R version 4.2.1 or higher

2. Required R packages (installed automatically if needed)

3. Minimal dataset file: FileS6_MinimalDataset_Complete.csv

ANALYSIS COMPONENTS:

-------------------

1. Data loading and verification

2. Descriptive statistics

3. Kaplan-Meier survival analysis

4. Cox proportional hazards models

5. Competing risks analysis (Fine-Gray)

6. Sensitivity analyses

7. Model diagnostics

8. Results export

HOW TO USE:

-----------

1. Place all files in the same directory

2. Open R or RStudio

3. Run: source("FileS5_Complete_Analysis_Code.R")

4. Check 'output' folder for results

OUTPUT STRUCTURE:

----------------

output/

├── figures/

│ ├── km_plot_consanguinity.png

│ └── martingale_residuals.png

├── tables/

│ ├── descriptive_table.csv

│ └── forest_plot_data.csv

└── analysis_results.rds (complete results)

NOTES:

------

- This code uses a minimal dataset of 12 representative cases

- Full analysis in the paper uses 3,427 cases

- All results are reproducible and transparent

- Contact researchers for full dataset access

CONTACT:

-------

For questions: n.taleb@ust.edu

============================================================================
