# Supplementary material for: Survival disparities and competing mortality risks in offspring of consanguineous marriages in Yemen: A 26-year retrospective cohort analysis
Source: PLoS One. 2026 May 29;21(5):e0349764. doi: 10.1371/journal.pone.0349764 (PMC13221058; doi:10.1371/journal.pone.0349764)
Supplement: S12 File — Complete validation studies, misclassification analysis, and quality assurance reports. (DOCX) [file pone.0349764.s012.docx]

# File S12: QUALITY CONTROL REPORTS

## EXECUTIVE SUMMARY

This document consolidates all quality control and assurance reports for the study:

"Survival Disparities and Competing Mortality Risks in Offspring of Consanguineous Marriages in Yemen: A 26-Year Retrospective Cohort Analysis."

All quality control procedures were implemented according to the pre-specified protocol, with continuous monitoring by the Data Monitoring Committee and Institutional Review Board. Key quality metrics met or exceeded established thresholds throughout the study period.

## 1. QUALITY ASSURANCE FRAMEWORK

### 1.1 Pre-Data Collection Quality Control

**Staff Training and Certification:**

- 2-week intensive training program completed by all field staff
- 100% certification rate achieved
- Inter-rater reliability: κ = 0.84 (exceeds 0.80 threshold)
- 10 supervised practice interviews per interviewer

**Instrument Testing and Validation:**

- 50-household pilot study conducted
- Cognitive testing of all questionnaire items
- Translation-back translation validation (Arabic-English)
- Cultural adaptation review by local experts and Community Advisory Board

### 1.2 During Data Collection Monitoring

**Field Supervision Protocol:**

- Daily supervisor spot-checking (10% of all interviews)
- Random back-checking within 48 hours of interview
- GPS verification of interview locations
- Audio recording review (10% random selection)

**Real-Time Quality Monitoring:**

- Electronic data validation with range and logic checks
- Missing data tracking with immediate follow-up
- Weekly data quality review meetings
- Quarterly quality metric reports to PI and DMC

### 1.3 Post-Data Collection Quality Assessment

**Data Validation Procedures:**

- Double data entry for 20% of forms (exceeds 10% threshold)
- Cross-source validation (medical records vs. interviews)
- Temporal consistency analysis across multiple interviews
- Expert panel review for complex cases (n=96, 15% of deaths)

**Quality Metrics Achieved:**

- Interview completion rate: 98.2%
- Item missing rate: <2%
- Inter-rater reliability: κ = 0.84
- Test-retest reliability: ICC = 0.91
- Medical record concordance: 84.2%

## 2. DATA MONITORING COMMITTEE REPORTS

### 2.1 DMC Composition and Charter

- Chair: Independent Epidemiologist (external institution)
- Statistician/Biostatistician
- Pediatric Geneticist
- Bioethicist
- Community Representative
- Meeting Frequency: Semi-annually

### 2.2 Meeting Summaries and Recommendations

**First Meeting (July 2023):**

- Reviewed study start-up procedures ✓ APPROVED
- Approved all data collection instruments ✓ APPROVED
- Confirmed safety monitoring plan ✓ APPROVED
- Recommendation: Proceed with data collection

**Second Meeting (January 2024):**

- Reviewed 6-month progress report
- Confirmed data quality metrics met thresholds
- No safety concerns identified
- Recommendation: Continue without modification

**Third Meeting (July 2024):**

- Reviewed interim data (first 1,500 participants)
- Confirmed protocol adherence
- Reviewed adverse event log (0 events)
- Recommendation: Continue to completion

**Overall DMC Assessment:**

"The study maintains high methodological rigor, participant safety, and data quality throughout all phases. No modifications or interruptions are warranted."

## 3. DATA QUALITY METRICS AND VALIDATION

### 3.1 Completeness and Accuracy

| **Metric** | **Result** | **Threshold** | **Status** |
| --- | --- | --- | --- |
| :--- | :--- | :--- | :--- |
| Interview completion rate | 98.2% | 95% | ✓ PASS |
| Item missing rate | 2.3% | <5% | ✓ PASS |
| Double data entry error rate | 0.8% | <2% | ✓ PASS |
| Medical record concordance | 84.2% | 80% | ✓ PASS |
| Temporal consistency | 96.7% | 95% | ✓ PASS |
| Cross-source validation | 92.4% | 90% | ✓ PASS |

### 3.2 Biological Plausibility Checks

- Age at death distribution: Consistent, no outliers ✓
- Birth year range (1998-2024): Logically consistent ✓
- Sex ratio (M:F = 1.05): Within normal range (0.9-1.1) ✓
- Follow-up dates > birth dates: 100% consistent ✓
- Consanguinity hierarchy validation: 100% consistent ✓

### 3.3 Verbal Autopsy Validation

**Validation Sub-study (n=96 deaths with medical records):**

- Physician-coded VA vs. hospital diagnosis concordance: 84.2%
- Cohen's κ: 0.79 (95% CI: 0.71-0.87)
- Physician inter-coder agreement: 91.3%
- Confidence level distribution: High (68%), Medium (27%), Low (5%)

**Corrective Actions:**

- Consensus review for uncertain cases (15% of deaths)
- Probabilistic bias analysis implemented
- Cause-grouping for competing risks analysis

## 4. STATISTICAL QUALITY CONTROL

### 4.1 Model Assumption Validation

| **Assumption** | **Test/Method** | **Result** | **Interpretation** | **Status** |
| --- | --- | --- | --- | --- |
| :--- | :--- | :--- | :--- | :--- |
| Proportional Hazards | Schoenfeld residuals (global) | χ²=8.34, p=0.134 | No significant violation | ✓ VALID |
| Linearity | Martingale residuals | p=0.234 | Linear form adequate | ✓ VALID |
| Influential Observations | Maximum DFBETA | 0.12 | <0.2 threshold | ✓ VALID |
| Multicollinearity | Maximum VIF | 2.34 | <5.0 threshold | ✓ VALID |
| Model Fit | Cox-Snell residuals | R²=0.342 | Adequate fit | ✓ VALID |
| Discrimination | Harrell's C-statistic | 0.79 (0.76-0.82) | Good discrimination | ✓ VALID |
| Calibration | Calibration slope | 1.02 (0.95-1.09) | Well calibrated | ✓ VALID |

### 4.2 Sensitivity Analysis Completeness

All pre-specified sensitivity analyses were completed:

1. Missing Data Handling: Multiple imputation (20 datasets), pattern mixture models

2. Time Scale Variations: Age-based, calendar time, time since diagnosis

3. Competing Risks Methods: Fine-Gray vs. cause-specific hazards

4. Model Specification: Full, reduced, Lasso-penalized models

5. Confounding Control: E-value assessment, propensity score adjustment

6. Diagnostic Accuracy: Probabilistic bias analysis (5%, 10%, 15% misclassification)

Conclusion: Primary results were robust across all sensitivity scenarios.

## 5. MONITORING AND AUDITING

### 5.1 Internal Monitoring Reports

- Monitor: Independent researcher not involved in study
- Frequency: Quarterly (Year 1), then semi-annually

**Areas Monitored:**

- Protocol adherence
- Consent process documentation
- Data quality and completeness
- Privacy and security procedures
- Safety reporting

Monitoring Reports Completed: 3

Overall Assessment: All satisfactory, no major deviations

### 5.2 External Audit Preparedness

**Documentation Available for Audit:**

- Complete protocol and all amendments
- All IRB correspondence and approvals
- Signed consent forms (100% available)
- Data management and security protocols
- Monitoring and safety reports
- Financial disclosure statements

**Audit Trail:**

- Version-controlled documents
- Time-stamped data entries
- Electronic signature logs
- Access and modification logs

### 5.3 Regulatory Compliance Verification

| **Standard/Guideline** | **Compliance Status** | **Verification** |
| --- | --- | --- |
| :--- | :--- | :--- |
| Declaration of Helsinki (2013) | ✓ FULLY COMPLIANT | File S14, S16 |
| CIOMS Guidelines (2016) | ✓ FULLY COMPLIANT | File S14, S16 |
| Yemeni National Health Research Guidelines | ✓ FULLY COMPLIANT | File S15 |
| STROBE/RECORD Guidelines | ✓ FULLY COMPLIANT | File S1 |
| FAIR Data Principles | ✓ FULLY COMPLIANT | All files |
| Conflict-affected Research Ethics | ✓ FULLY COMPLIANT | File S16 |

## 6. SAFETY AND RISK MANAGEMENT REPORTS

### 6.1 Adverse Event Monitoring

- Reporting Period: April 2023 - December 2024
- Total Families Recruited: 1,065 (representing 3,427 offspring)
- Serious Adverse Events: 0
- Non-Serious Adverse Events: 12 (1.1% of participants)
- All psychological distress related to bereavement discussion
- All resolved with counseling support (average 2.3 sessions)
- No study discontinuations due to adverse events

### 6.2 Risk Mitigation Effectiveness

| **Risk** | **Mitigation Strategy** | **Effectiveness** |
| --- | --- | --- |
| :--- | :--- | :--- |
| Psychological distress | Trained interviewers, counseling referrals | 100% resolution |
| Confidentiality breach | Immediate anonymization, encryption | 0 breaches |
| Stigma related to consanguinity | Community sensitization, CAB involvement | No incidents |
| Physical safety in conflict zones | Security protocols, travel restrictions | 0 safety incidents |

### 6.3 Conflict-Affected Setting Adaptations

**Data Collection During Instability (2015-2019):**

- Temporary suspension during peak conflict months
- Enhanced verification procedures implemented:
- Triple-source verification for vital status
- Longitudinal consistency checks
- Conservative classification of uncertain cases
- Sensitivity analysis confirmed robustness of results

**Healthcare Access Measurement Adaptation:**

- Modified composite score during facility closures
- Focus on travel time to nearest functioning facility
- Essential medication availability assessment

## 7. DATA SECURITY AND PRIVACY REPORTS

### 7.1 Data Protection Impact Assessment

- Assessment Date: 10 March 2023

**Data Categories Processed:**

- Health data (special category) ✓ Protected
- Genetic information (special category) ✓ Protected
- Family relationship data ✓ Protected
- Socioeconomic information ✓ Protected

**Protection Measures Implemented:**

- Pseudonymization at point of collection ✓
- AES-256 encryption at rest and in transit ✓
- Two-factor authentication for access ✓
- Regular security audits (quarterly) ✓
- Access logging with anomaly detection ✓

### 7.2 Privacy by Design Implementation

- Data Minimization: Only essential data collected ✓
- Purpose Limitation: Data used only for specified research ✓
- Storage Limitation: Retention for 10 years post-study ✓
- Access Controls: Role-based (3 levels) with regular review ✓

### 7.3 Data Breach Monitoring

- Monitoring Period: April 2023 - December 2024
- Data Breaches: 0
- Security Incidents: 0
- Access Anomalies Detected: 3 (all investigated, none malicious)

## 8. COMMUNITY ENGAGEMENT QUALITY REPORTS

### 8.1 Community Advisory Board (CAB) Activities

- Establishment: April 2023
- Membership: 11 members (community elders, religious leaders, healthcare workers, parents, women's representatives)
- Meeting Frequency: Quarterly

**Key Contributions:**

- Cultural adaptation of consent procedures ✓
- Input on sensitive question wording ✓
- Community entry strategy development ✓
- Feedback dissemination planning ✓

### 8.2 Community Feedback Mechanism

**Channels Available:**

- Dedicated phone line (23 calls received)
- Community meetings (4 conducted)
- Suggestion boxes at health centers

**Feedback Received and Addressed:**

- Total concerns: 23 (all resolved)
- Most common: Request for study results in lay language
- Response: Commitment to community feedback sessions post-publication

### 8.3 Cultural Adaptation Effectiveness

**Consent Process Adaptations:**

- Witnessed thumbprint consent: 100% implementation for illiterate participants
- Family-based consent where appropriate: Applied in 34% of cases
- Extended consent process for sensitive topics: Average 45 minutes

**Interview Protocol Adaptations:**

- Gender-matched interviewers available: 100% on request
- Private interview spaces: 100% secured

## 9. OVERALL QUALITY ASSESSMENT AND CONCLUSIONS

### 9.1 Summary of Quality Achievements

1. **Methodological Rigor:** All pre-specified protocols followed, sensitivity analyses completed

2. **Data Quality:** All metrics met or exceeded thresholds, validation studies confirm accuracy

3. **Participant Safety:** No serious adverse events, effective risk mitigation

4. **Ethical Compliance:** Full adherence to national and international standards

5. **Statistical Validity:** Model assumptions met, robust to sensitivity testing

6. **Transparency and Reproducibility:** Complete documentation and code provided

### 9.2 Limitations and Corrective Actions

| **Limitation** | **Corrective Action** | **Effectiveness** |
| --- | --- | --- |
| :--- | :--- | :--- |
| Verbal autopsy limitations | Consensus review, probabilistic bias analysis | ✓ Effective |
| Diagnostic evolution over 26 years | Re-review using current criteria, sensitivity analysis | ✓ Effective |
| Conflict-affected data collection | Enhanced verification, temporal sensitivity analysis | ✓ Effective |
| Geographic generalizability | Clear contextualization, comparison with regional data | ✓ Addressed |

### 9.3 Recommendations for Future Research

Based on quality control findings:

1. Prospective validation of competing risks models recommended

2. Integration of genomic data would enhance precision

3. Dynamic prediction models with time-varying covariates suggested

4. Quality-of-life-adjusted survival analysis recommended for comprehensive assessment

## 10. SIGNATURES AND CERTIFICATIONS

### 10.1 Principal Investigator Certification

I certify that all quality control procedures documented in this report were implemented as described, and that the study maintains the highest standards of scientific rigor, ethical conduct, and participant protection throughout its duration.

Signature: ___________________________

Naif Taleb Ali, Principal Investigator

Date: //_______

### 10.2 Data Monitoring Committee Endorsement

The Data Monitoring Committee has reviewed all quality control reports and confirms that the study maintains appropriate quality standards and may continue/be considered complete as conducted.

Signature: ___________________________

Chair, Independent Data Monitoring Committee

Date: //_______

### 10.3 Institutional Review Board Acknowledgment

The IRB acknowledges receipt and review of these quality control reports as part of the continuing review process.

Signature: ___________________________

Dr. Adel Ahmed, Chairperson

Radfan College University IRB

Date: //_______

## APPENDICES

- Appendix A: Monthly Quality Metric Reports (Available electronically)
- Appendix B: Data Monitoring Committee Meeting Minutes (Available electronically)
- Appendix C: Validation Study Raw Data (Available upon request)
- Appendix D: Security Audit Reports (Available electronically - restricted access)
- Appendix E: Community Feedback Log (Available upon request)

**Document Version:** 1.0

**Last Updated:** December 2024

**Contact:** Naif Taleb Ali (n.taleb@ust.edu)

**Study Reference:** RUC-IRB-2023-045 / AMREC 2024-011
