# Supplementary material for: Survival disparities and competing mortality risks in offspring of consanguineous marriages in Yemen: A 26-year retrospective cohort analysis
Source: PLoS One. 2026 May 29;21(5):e0349764. doi: 10.1371/journal.pone.0349764 (PMC13221058; doi:10.1371/journal.pone.0349764)
Supplement: S1 Table — (DOCX) [file pone.0349764.s013.docx]

**Table S1: Complete Cohort Characteristics by Vital Status**

| Characteristic | Total Cohort | Survivors | Deceased | p-value |
| --- | --- | --- | --- | --- |
| Male | 1,756 (51.2%) | 1,387 (49.7%) | 369 (57.8%) | 0.001 |
| Female | 1,671 (48.8%) | 1,402 (50.3%) | 269 (42.2%) | 0.001 |
| 1998-2002 | 845 (24.7%) | 623 (22.3%) | 222 (34.8%) | <0.001 |
| 2003-2007 | 967 (28.2%) | 778 (27.9%) | 189 (29.6%) | 0.38 |
| 2008-2012 | 892 (26.0%) | 756 (27.1%) | 136 (21.3%) | 0.004 |
| 2013-2024 | 723 (21.1%) | 632 (22.7%) | 91 (14.3%) | <0.001 |
| First cousins | 1,243 (36.3%) | 923 (33.1%) | 320 (50.2%) | <0.001 |
| Other consanguineous | 723 (21.1%) | 612 (21.9%) | 111 (17.4%) | 0.024 |
| Non-consanguineous | 1,461 (42.6%) | 1,254 (45.0%) | 207 (32.4%) | <0.001 |
