# Supplementary material for: Survival disparities and competing mortality risks in offspring of consanguineous marriages in Yemen: A 26-year retrospective cohort analysis
Source: PLoS One. 2026 May 29;21(5):e0349764. doi: 10.1371/journal.pone.0349764 (PMC13221058; doi:10.1371/journal.pone.0349764)
Supplement: S2 Table — (DOCX) [file pone.0349764.s014.docx]

**Table S2: Complete Cause-Specific Mortality Analysis by Age Groups**

| Age Group | Cause of Death | n | Cumulative Incidence at 5 Years (95% CI) | Subdistribution HR (95% CI) | Median Age at Death (months) |
| --- | --- | --- | --- | --- | --- |
| <1 Year | Congenital anomalies | 134 | 24.3% (21.8-27.1) | 5.8 (3.4-9.9) | 3.2 [1.4-6.7] |
| <1 Year | Cardiac defects | 78 | 12.7% (10.8-14.9) | 8.9 (5.2-15.3) | 2.8 [1.2-5.1] |
| <1 Year | Multiple anomalies | 56 | 8.9% (7.3-10.8) | 6.3 (3.5-11.4) | 4.3 [2.1-7.8] |
| 1-5 Years | Hematological disorders | 167 | 18.7% (16.5-21.1) | 4.3 (2.6-7.1) | 38.4 [24.7-52.1] |
| 1-5 Years | β-thalassemia complications | 112 | 12.4% (10.6-14.5) | 7.2 (4.1-12.6) | 32.7 [18.9-47.2] |
| 1-5 Years | Infection-related | 45 | 4.2% (3.2-5.5) | 2.8 (1.4-5.6) | 41.2 [28.3-55.6] |
| 5-15 Years | Neurological complications | 98 | 12.4% (10.6-14.5) | 2.9 (1.7-4.9) | 108.7 [84.3-132.4] |
| 5-15 Years | Epilepsy-related | 56 | 7.8% (6.4-9.5) | 3.4 (1.9-6.1) | 112.5 [89.1-138.2] |
| 5-15 Years | Neurodegenerative | 34 | 3.2% (2.4-4.3) | 2.1 (1.1-4.2) | 126.8 [102.3-151.9] |
| >15 Years | Multisystem failure | 45 | 8.9% (7.3-10.8) | 2.1 (1.2-3.7) | 212.4 [187.6-254.3] |
