# Supplementary material for: Survival disparities and competing mortality risks in offspring of consanguineous marriages in Yemen: A 26-year retrospective cohort analysis
Source: PLoS One. 2026 May 29;21(5):e0349764. doi: 10.1371/journal.pone.0349764 (PMC13221058; doi:10.1371/journal.pone.0349764)
Supplement: S3 Table — (DOCX) [file pone.0349764.s015.docx]

**Table S3: Multivariable Cox Regression Full Results**

| Predictor | Level | Hazard Ratio | 95% CI | p-value |
| --- | --- | --- | --- | --- |
| Consanguinity | First cousins | 2.84 | 2.32-3.44 | <0.001 |
| Consanguinity | Second cousins | 2.12 | 1.67-2.62 | <0.001 |
| Consanguinity | Beyond second cousins | 1.42 | 1.08-1.78 | 0.012 |
| Disorder type | Hematological | 8.42 | 5.23-13.56 | <0.001 |
| Disorder type | Congenital anomalies | 4.23 | 2.84-6.32 | <0.001 |
| Disorder type | Neurodevelopmental | 2.12 | 1.42-3.21 | <0.001 |
| Sex | Male | 1.34 | 1.12-1.61 | 0.001 |
| Residence | Rural | 1.78 | 1.42-2.33 | <0.001 |
| Parental education | Illiterate | 1.84 | 1.45-2.34 | <0.001 |
| Parental education | Primary | 1.56 | 1.23-1.98 | <0.001 |
| Parental education | Secondary | 1.23 | 0.98-1.54 | 0.074 |
| Birth cohort | 1998-2002 | 2.34 | 1.87-2.93 | <0.001 |
| Birth cohort | 2003-2007 | 1.89 | 1.52-2.35 | <0.001 |
| Birth cohort | 2008-2012 | 1.45 | 1.16-1.81 | 0.001 |
