# Supplementary material for: Survival disparities and competing mortality risks in offspring of consanguineous marriages in Yemen: A 26-year retrospective cohort analysis
Source: PLoS One. 2026 May 29;21(5):e0349764. doi: 10.1371/journal.pone.0349764 (PMC13221058; doi:10.1371/journal.pone.0349764)
Supplement: S4 Table — (DOCX) [file pone.0349764.s016.docx]

**Table S4: Fine-Gray Competing Risks Complete Output**

| Cause of Death | Predictor | Subdistribution HR | 95% CI | p-value | Cumulative Incidence at 10 Years |
| --- | --- | --- | --- | --- | --- |
| Congenital anomalies | First cousins | 3.62 | 2.56-5.12 | <0.001 | 26.7% (24.1-29.4) |
| Congenital anomalies | Hematological disorders | 1.23 | 0.87-1.74 | 0.245 |  |
| Congenital anomalies | Rural residence | 1.89 | 1.34-2.67 | <0.001 |  |
| Hematological disorders | First cousins | 4.73 | 3.34-6.70 | <0.001 | 23.4% (21.2-25.8) |
| Hematological disorders | β-thalassemia major | 7.21 | 4.87-10.67 | <0.001 |  |
| Hematological disorders | Low parental education | 2.34 | 1.67-3.28 | <0.001 |  |
| Neurodevelopmental | First cousins | 2.34 | 1.63-3.36 | <0.001 | 15.6% (13.8-17.6) |
| Neurodevelopmental | Severe intellectual disability | 3.45 | 2.34-5.09 | <0.001 |  |
| Neurodevelopmental | Rural residence | 1.67 | 1.18-2.36 | 0.004 |  |
