# Supplementary material for: Survival disparities and competing mortality risks in offspring of consanguineous marriages in Yemen: A 26-year retrospective cohort analysis
Source: PLoS One. 2026 May 29;21(5):e0349764. doi: 10.1371/journal.pone.0349764 (PMC13221058; doi:10.1371/journal.pone.0349764)
Supplement: S5 Table — (DOCX) [file pone.0349764.s017.docx]

**Table S5: Temporal Survival Trends by Birth Cohort**

| Birth Cohort | Hemoglobinopathies | Congenital Anomalies | Neurodevelopmental | Sensory Impairments | Overall Mortality Reduction |
| --- | --- | --- | --- | --- | --- |
| 1998-2002 | 28.9% | 54.2% | 69.8% | 88.9% | Reference |
| 2003-2007 | 37.4% | 61.7% | 75.6% | 90.2% | 23.4% |
| 2008-2012 | 48.2% | 70.3% | 82.9% | 91.7% | 41.7% |
| 2013-2024 | 62.7% | 78.9% | 87.4% | 93.4% | 58.9% |
| p-trend | <0.001 | <0.001 | <0.001 | 0.012 | <0.001 |
