# Supplementary material for: Survival disparities and competing mortality risks in offspring of consanguineous marriages in Yemen: A 26-year retrospective cohort analysis
Source: PLoS One. 2026 May 29;21(5):e0349764. doi: 10.1371/journal.pone.0349764 (PMC13221058; doi:10.1371/journal.pone.0349764)
Supplement: S6 Table — (DOCX) [file pone.0349764.s018.docx]

**Table S6: Model Diagnostics and Validation Summary**

| Diagnostic Test | Statistic | p-value | Interpretation |
| --- | --- | --- | --- |
| Global test | χ²=8.34 | 0.134 | Assumption satisfied |
| Consanguinity | χ²=2.45 | 0.485 | Assumption satisfied |
| Disorder type | χ²=3.67 | 0.298 | Assumption satisfied |
| Maximum DFBETA | 0.12 | - | No concerning influence |
| Observations with DFBETA >0.1 | 23 (0.7%) | - |  |
| Martingale residuals | - | 0.234 | Linear form adequate |
| Cox-Snell residuals | R²=0.342 | - | Adequate fit |
| Maximum VIF | 2.34 | - | No concerning collinearity |
