# Supplementary material for: Survival disparities and competing mortality risks in offspring of consanguineous marriages in Yemen: A 26-year retrospective cohort analysis
Source: PLoS One. 2026 May 29;21(5):e0349764. doi: 10.1371/journal.pone.0349764 (PMC13221058; doi:10.1371/journal.pone.0349764)
Supplement: S7 Table — (DOCX) [file pone.0349764.s019.docx]

**Table S7: Sensitivity Analysis Results**

| Analysis Type | Consanguinity HR (95% CI) | Disorder Type HR (95% CI) | Conclusion |
| --- | --- | --- | --- |
| Primary Analysis | 2.84 (2.32-3.44) | 8.42 (5.23-13.56) | Reference |
| Multiple Imputation | 2.79 (2.28-3.41) | 8.35 (5.18-13.47) | Robust |
| Landmark (1 year) | 2.91 (2.35-3.60) | 8.56 (5.31-13.80) | Robust |
| Cause-Specific Hazards | 2.88 (2.36-3.52) | 8.48 (5.27-13.65) | Robust |
| Complete Cases | 2.81 (2.29-3.45) | 8.38 (5.20-13.51) | Robust |
| Alternative PH Model | 2.86 (2.34-3.49) | 8.45 (5.25-13.61) | Robust |
