# Supplementary material for: Survival disparities and competing mortality risks in offspring of consanguineous marriages in Yemen: A 26-year retrospective cohort analysis
Source: PLoS One. 2026 May 29;21(5):e0349764. doi: 10.1371/journal.pone.0349764 (PMC13221058; doi:10.1371/journal.pone.0349764)
Supplement: S8 Table — (DOCX) [file pone.0349764.s020.docx]

**Table S8: Subgroup Analysis by Geographic Region**

| Region | n | Consanguinity HR (95% CI) | Hematological Disorders HR (95% CI) | 5-Year Survival | CHE Incidence |
| --- | --- | --- | --- | --- | --- |
| Urban Central | 623 | 2.45 (1.87-3.21) | 7.89 (4.56-13.65) | 78.9% | 34.2% |
| Urban Peripheral | 381 | 2.67 (1.98-3.60) | 8.23 (4.78-14.17) | 76.4% | 38.9% |
| Rural Accessible | 1,234 | 2.92 (2.28-3.74) | 8.56 (5.12-14.31) | 72.3% | 52.7% |
| Rural Remote | 1,189 | 3.12 (2.45-3.97) | 8.78 (5.27-14.63) | 68.9% | 67.8% |
| p-interaction |  | 0.045 | 0.287 | <0.001 | <0.001 |
