# Supplementary material for: Survival disparities and competing mortality risks in offspring of consanguineous marriages in Yemen: A 26-year retrospective cohort analysis
Source: PLoS One. 2026 May 29;21(5):e0349764. doi: 10.1371/journal.pone.0349764 (PMC13221058; doi:10.1371/journal.pone.0349764)
Supplement: S9 Table — (DOCX) [file pone.0349764.s021.docx]

**Table S9: Economic Correlates of Survival Outcomes**

| Economic Indicator | Level | Hazard Ratio (95% CI) | 5-Year Survival | CHE Incidence |
| --- | --- | --- | --- | --- |
| Household Income | Highest tertile | 1.00 (Ref) | 82.3% | 23.4% |
| Household Income | Middle tertile | 1.45 (1.12-1.87) | 76.7% | 45.6% |
| Household Income | Lowest tertile | 2.34 (1.84-2.98) | 67.8% | 78.9% |
| Health Insurance | Covered | 1.00 (Ref) | 84.5% | 18.9% |
| Health Insurance | Not covered | 1.89 (1.48-2.41) | 71.2% | 62.7% |
| Distance to Hospital | <5 km | 1.00 (Ref) | 81.6% | 28.9% |
| Distance to Hospital | 5-15 km | 1.56 (1.22-2.00) | 74.3% | 47.8% |
| Distance to Hospital | >15 km | 2.23 (1.75-2.84) | 65.1% | 72.3% |
