# Supplementary material for: Survival disparities and competing mortality risks in offspring of consanguineous marriages in Yemen: A 26-year retrospective cohort analysis
Source: PLoS One. 2026 May 29;21(5):e0349764. doi: 10.1371/journal.pone.0349764 (PMC13221058; doi:10.1371/journal.pone.0349764)
Supplement: S10 Table — (DOC) [file pone.0349764.s022.docx]

**Table S10: Quality of Life and Functional Outcomes**

| Domain | Affected Children Mean ± SD | Healthy Siblings Mean ± SD | Difference (95% CI) | p-value |
| --- | --- | --- | --- | --- |
| Physical Functioning | 52.3 ± 18.4 | 84.5 ± 12.3 | -32.2 (-34.1 to -30.3) | <0.001 |
| Emotional Functioning | 45.6 ± 20.1 | 82.3 ± 13.4 | -36.7 (-38.9 to -34.5) | <0.001 |
| Social Functioning | 38.9 ± 22.3 | 86.7 ± 11.2 | -47.8 (-50.2 to -45.4) | <0.001 |
| School Functioning | 41.2 ± 19.8 | 81.9 ± 14.5 | -40.7 (-42.8 to -38.6) | <0.001 |
| Psychosocial Summary | 41.9 ± 19.4 | 83.6 ± 12.1 | -41.7 (-43.6 to -39.8) | <0.001 |
| Total Score | 45.2 ± 18.7 | 84.1 ± 11.9 | -38.9 (-40.7 to -37.1) | <0.001 |
