# Supplementary material for: Survival disparities and competing mortality risks in offspring of consanguineous marriages in Yemen: A 26-year retrospective cohort analysis
Source: PLoS One. 2026 May 29;21(5):e0349764. doi: 10.1371/journal.pone.0349764 (PMC13221058; doi:10.1371/journal.pone.0349764)
Supplement: S11 Table — (DOCX) [file pone.0349764.s023.docx]

**Table S11: Complete Sampling Framework**

| Sampling Stage | Description | Clusters | Households |
| --- | --- | --- | --- |
| Stage 1: Stratification | Urban vs Rural division | 12 strata | - |
| Stage 2: Cluster sampling | Random selection of villages | 45 clusters | - |
| Stage 3: Household enumeration | Complete listing of eligible households | - | 1,580 |
| Stage 4: Verification | Reproductive history validation | - | 1,327 eligible |
