# Supplementary material for: Survival disparities and competing mortality risks in offspring of consanguineous marriages in Yemen: A 26-year retrospective cohort analysis
Source: PLoS One. 2026 May 29;21(5):e0349764. doi: 10.1371/journal.pone.0349764 (PMC13221058; doi:10.1371/journal.pone.0349764)
Supplement: S12 Table — (DOCX) [file pone.0349764.s024.docx]

**Table S12: Power Analysis Scenarios**

| Scenario | Sample Size | Hazard Ratio | Event Rate | Statistical Power |
| --- | --- | --- | --- | --- |
| Main effect | 1,065 | 2.0 | 18% | 92% |
| Interaction effect | 1,065 | 1.8 | 18% | 85% |
| Subgroup analysis | 532 | 2.2 | 18% | 78% |
| Rare outcome | 1,065 | 2.5 | 8% | 76% |
