# Supplementary material for: Survival disparities and competing mortality risks in offspring of consanguineous marriages in Yemen: A 26-year retrospective cohort analysis
Source: PLoS One. 2026 May 29;21(5):e0349764. doi: 10.1371/journal.pone.0349764 (PMC13221058; doi:10.1371/journal.pone.0349764)
Supplement: S13 Table — (DOCX) [file pone.0349764.s025.docx]

**Table S13: Prediction Model Performance**

| Model | C-statistic | Brier Score | Calibration Slope |
| --- | --- | --- | --- |
| Basic Cox model | 0.76 | 0.18 | 0.98 |
| Enhanced with interactions | 0.79 | 0.16 | 1.02 |
| Risk score model | 0.74 | 0.19 | 0.95 |
