# Supplementary material for: Survival disparities and competing mortality risks in offspring of consanguineous marriages in Yemen: A 26-year retrospective cohort analysis
Source: PLoS One. 2026 May 29;21(5):e0349764. doi: 10.1371/journal.pone.0349764 (PMC13221058; doi:10.1371/journal.pone.0349764)
Supplement: S14 Table — (DOCX) [file pone.0349764.s026.docx]

**Table S14: Disease Burden by Disorder Type**

| Disorder Type | DALYs | YLL | YLD | Economic Burden |
| --- | --- | --- | --- | --- |
| Hematological | 4,890 | 3,450 | 1,440 | $24.45M |
| Congenital | 3,780 | 2,890 | 890 | $18.90M |
| Neurodevelopmental | 2,340 | 1,230 | 1,110 | $11.70M |
| Sensory | 890 | 340 | 550 | $4.45M |
| Other | 550 | 280 | 270 | $2.75M |
