# Supplementary material for: Survival disparities and competing mortality risks in offspring of consanguineous marriages in Yemen: A 26-year retrospective cohort analysis
Source: PLoS One. 2026 May 29;21(5):e0349764. doi: 10.1371/journal.pone.0349764 (PMC13221058; doi:10.1371/journal.pone.0349764)
Supplement: S15 Table — (DOCX) [file pone.0349764.s027.docx]

**Table S15: Cost-Effectiveness of Interventions**

| Intervention Strategy | Cost per Family | DALYs Averted | Incremental Cost | ICER |
| --- | --- | --- | --- | --- |
| No screening | $0 | 0 | - | - |
| Targeted screening | $500 | 1,500 | $500 | $334 |
| Universal screening | $1,000 | 2,500 | $500 | $500 |
