# Supplementary material for: Survival disparities and competing mortality risks in offspring of consanguineous marriages in Yemen: A 26-year retrospective cohort analysis
Source: PLoS One. 2026 May 29;21(5):e0349764. doi: 10.1371/journal.pone.0349764 (PMC13221058; doi:10.1371/journal.pone.0349764)
Supplement: S16 Table — (DOCX) [file pone.0349764.s028.docx]

**Table S16: Sensitivity Analysis for Cause of Death Misclassification**

| Scenario | Consanguinity HR (Corrected) | 95% CI | Congenital Anomalies HR (Corrected) | 95% CI |
| --- | --- | --- | --- | --- |
| Base Case | 2.84 | 2.32-3.44 | 4.23 | 2.84-6.32 |
| 5% Misclassification | 2.79 | 2.28-3.41 | 4.18 | 2.79-6.26 |
| 10% Misclassification | 2.75 | 2.25-3.36 | 4.12 | 2.75-6.18 |
| 15% Misclassification | 2.70 | 2.21-3.30 | 4.05 | 2.70-6.07 |
| Differential Correction | 2.81 | 2.30-3.43 | 4.20 | 2.81-6.28 |
