# Supplementary material for: Survival disparities and competing mortality risks in offspring of consanguineous marriages in Yemen: A 26-year retrospective cohort analysis
Source: PLoS One. 2026 May 29;21(5):e0349764. doi: 10.1371/journal.pone.0349764 (PMC13221058; doi:10.1371/journal.pone.0349764)
Supplement: S17 Table — (DOCX) [file pone.0349764.s029.docx]

**Table S17: Interaction Effects Between Key Predictors**

| Interaction Term | Hazard Ratio | 95% CI | p-interaction |
| --- | --- | --- | --- |
| Consanguinity × Healthcare Access | 1.45 | 1.18-1.78 | 0.001 |
| Disorder Severity × Birth Cohort | 0.76 | 0.63-0.92 | 0.005 |
| Rural Residence × Parent Education | 1.62 | 1.29-2.03 | <0.001 |
| Consanguinity × Disorder Type | 1.83 | 1.45-2.31 | <0.001 |
