# Supplementary material for: Survival disparities and competing mortality risks in offspring of consanguineous marriages in Yemen: A 26-year retrospective cohort analysis
Source: PLoS One. 2026 May 29;21(5):e0349764. doi: 10.1371/journal.pone.0349764 (PMC13221058; doi:10.1371/journal.pone.0349764)
Supplement: S18 Table — (DOCX) [file pone.0349764.s030.docx]

**Table S18: Minimal Dataset Case Description**

| **Case ID** | **Birth Year** | **Sex** | **Consanguinity** | **Disorder Type** | **Vital Status** | **Age at Death** | **Purpose in Minimal Dataset** |
| --- | --- | --- | --- | --- | --- | --- | --- |
| 1 | 2000 | Male | First cousins | Hematological | Deceased | 4.2 years | Represents early childhood mortality from hematological disorder |
| 2 | 2005 | Female | Non-consanguineous | Sensory impairment | Alive | NA | Represents non-fatal disorder in recent cohort |
| 3 | 2010 | Male | Second cousins | Congenital anomaly | Deceased | 0.8 years | Represents infant mortality from structural anomaly |
| 4 | 2015 | Female | First cousins | Neurodevelopmental | Alive | NA | Represents ongoing neurodevelopmental condition |
| 5 | 1999 | Male | Beyond second cousins | Hematological | Deceased | 7.5 years | Represents late childhood mortality from sickle cell disease |
| 6 | 2008 | Female | First cousins | Congenital anomaly | Deceased | 0.3 years | Represents very early infant mortality |
| 7 | 2012 | Male | Non-consanguineous | Sensory impairment | Alive | NA | Represents urban, educated family case |
| 8 | 2003 | Female | Second cousins | Neurodevelopmental | Alive | NA | Represents rural long-term survivor |
| 9 | 2018 | Male | First cousins | Hematological | Alive | NA | Represents recent birth with intermediate disorder |
| 10 | 2001 | Female | Non-consanguineous | No disorder | Alive | NA | Represents healthy control |
| 11 | 2006 | Male | Beyond second cousins | Congenital anomaly | Alive | NA | Represents non-fatal structural anomaly |
| 12 | 2014 | Female | First cousins | Neurodevelopmental | Deceased | 3.2 years | Represents mortality from complications |

*Purpose: The minimal dataset (12 cases) demonstrates data structure and variable coding while preserving key statistical relationships. Full analyses utilize the complete cohort of 3,427 offspring.*
