# Supplementary material for: Survival disparities and competing mortality risks in offspring of consanguineous marriages in Yemen: A 26-year retrospective cohort analysis
Source: PLoS One. 2026 May 29;21(5):e0349764. doi: 10.1371/journal.pone.0349764 (PMC13221058; doi:10.1371/journal.pone.0349764)
Supplement: S19 Table — (DOCX) [file pone.0349764.s031.docx]

**Table S19: Trends in Consanguinity by Birth Cohort**

| **Birth Cohort** | **First Cousins n (%)** | **Second Cousins n (%)** | **Beyond Second Cousins n (%)** | **Non-consanguineous n (%)** | **Total n** |
| --- | --- | --- | --- | --- | --- |
| 1998–2002 | 302 (35.8%) | 170 (20.1%) | 86 (10.2%) | 287 (33.9%) | 845 |
| 2003–2007 | 348 (36.0%) | 196 (20.3%) | 102 (10.5%) | 321 (33.2%) | 967 |
| 2008–2012 | 323 (36.2%) | 183 (20.5%) | 96 (10.8%) | 290 (32.5%) | 892 |
| 2013–2024 | 261 (36.1%) | 147 (20.4%) | 77 (10.7%) | 238 (32.8%) | 723 |
| **Total** | **1,234 (36.0%)** | **696 (20.3%)** | **361 (10.5%)** | **1,136 (33.2%)** | **3,427** |

**Consanguinity rates remained stable across the 26-year study period (χ² test for trend: p = 0.874).*
