# Supplementary material for: Survival disparities and competing mortality risks in offspring of consanguineous marriages in Yemen: A 26-year retrospective cohort analysis
Source: PLoS One. 2026 May 29;21(5):e0349764. doi: 10.1371/journal.pone.0349764 (PMC13221058; doi:10.1371/journal.pone.0349764)
Supplement: Attachment — Submitted filename: Point to point respone.pdf [file pone.0349764.s045.pdf]

Response to Journal Requirements and Reviewers' Comments

Manuscript ID: PONE-D-25-65515

Title: Survival Disparities and Competing Mortality Risks in Offspring of Consanguineous Marriages in Yemen: A 26-Year Retrospective Cohort Analysis

Dear Dr. Mainak Bardhan and Reviewers,

Thank you for considering our manuscript and for the constructive feedback from the reviewers. We have carefully addressed all comments and have revised the manuscript accordingly. Below is our point-by-point response.

Journal Requirements:

1. Style requirements: We have reformatted the manuscript according to PLOS ONE templates.

2. Ethics statement: We have moved the ethics statement to the Methods section only (under "Ethical Considerations and Research Conduct").

3. Figures S1–S13: We have re-uploaded all supplementary figures in TIFF format with high resolution (300 DPI minimum), converted using PLOS's recommended NAAS tool as suggested in the submission guidelines.

4. Map in Figure S13: We have completely removed Figure S13 (the map) from the supplementary materials to avoid any copyright issues, as we were unable to secure permission for the original map under CC BY 4.0 license.

5. References: We have reviewed and corrected the reference list. No retracted papers were cited. We have added relevant suggested references (Bener et al. 2019; Hamamy 2012; Bittles 2001).

6. Reviewer-suggested citations: We have evaluated and included relevant suggested references where appropriate.

Reviewer 1:

Comment 1.1: "Regarding the treatment of 'Healthcare Access' as a time-dependent covariate… there is a significant risk of reverse causality…"

Response: We thank the reviewer for this insightful observation. We have added a new subsection in the Methods titled "Residential Stability Assessment" where we clarify that 94.2% of families did not change residence after diagnosis. Sensitivity analyses excluding migrating families yielded consistent results (HR range: 2.81–2.85). We have also added a corresponding limitation in the Discussion.

Comment 1.2: "In your 'Policy Implications', you suggest expanding newborn screening. Given the current economic burden… is this feasible?"

Response: We have added a new paragraph in the Discussion under "Economic Feasibility in a Conflict-Affected Setting" where we provide a cost-per-case-detected estimate ($1,200) and note that the ICER of $334 per DALY averted is highly cost-effective. We also discuss phased implementation starting in urban centers.

Comment 1.3: "Did the rate of consanguineous marriage change over the 26-year study period?"

Response: We have added a new supplementary table (Table S19) showing trends in consanguinity by birth cohort. The practice remained stable across the 26-year period (first-cousin marriages: ~36% throughout).

Reviewer 2:

Comment 2.1: "The methodology… would benefit from a clearer explanation of how confounding variables were controlled…"

Response: We thank the reviewer for this valuable methodological suggestion. To address this, we have expanded the "Statistical Analysis Plan" subsection in the Methods to provide a clearer and more detailed explanation of our confounder adjustment strategy. Specifically, we now describe: (1) the use of directed acyclic graphs (DAGs) for covariate selection based on theoretical considerations and prior literature; (2) multi-variable adjustment with both time-independent and time-dependent covariates; and (3) sensitivity analyses to assess residual confounding. These additions enhance the transparency and robustness of our analytical approach.

Comment 2.2: "The discussion would be more comprehensive if it explicitly addressed barriers related to accessibility, affordability, and acceptability…"

Response: We have added a new paragraph in the Discussion under "Policy Implications and Health System Integration" addressing these barriers, citing relevant literature from similar settings (Pakistan, Sudan) and emphasizing culturally sensitive delivery channels.

Comment 2.3: "The authors should emphasize that prevention can begin even earlier—before marriage or before planning a pregnancy…"

Response: We have expanded the "Priority Action Plan" to include a "Pre-marital and pre-conception prevention" tier as the first step, citing successful examples from Qatar and Jordan (Bener et al. 2019; Hamamy 2012).

We believe these revisions have strengthened the manuscript significantly. Thank you for the opportunity to resubmit.

Sincerely,

Naif Taleb Ali, on behalf of all authors.
